# A Landscape-Scale, Applied Fire Management Experiment Promotes Recovery of a Population of the Threatened Gouldian Finch, *Erythrura gouldiae*, in Australia’s Tropical Savannas

**DOI:** 10.1371/journal.pone.0137997

**Published:** 2015-10-07

**Authors:** Sarah Legge, Stephen Garnett, Kim Maute, Joanne Heathcote, Steve Murphy, John C. Z. Woinarski, Lee Astheimer

**Affiliations:** 1 Australian Wildlife Conservancy, PO Box 8070, Subiaco East, WA, 6008, Australia; 2 Research Institute for the Environment and Livelihoods and Threatened Species Recovery Hub of the National Environmental Science Programme, Charles Darwin University, NT, 0909, Australia; 3 Institute of Conservation Biology and Environmental Management, University of Wollongong, Wollongong, NSW, 2522, Australia; 4 Bush Heritage Australia, Collins St, Melbourne, VIC, Australia; 5 Deakin University, DVC Research Office, Locked Bag 20000, Geelong, VIC, 3220, Australia; University of New South Wales, AUSTRALIA

## Abstract

Fire is an integral part of savanna ecology and changes in fire patterns are linked to biodiversity loss in savannas worldwide. In Australia, changed fire regimes are implicated in the contemporary declines of small mammals, riparian species, obligate-seeding plants and grass seed-eating birds. Translating this knowledge into management to recover threatened species has proved elusive. We report here on a landscape-scale experiment carried out by the Australian Wildlife Conservancy (AWC) on Mornington Wildlife Sanctuary in northwest Australia. The experiment was designed to understand the response of a key savanna bird guild to fire, and to use that information to manage fire with the aim of recovering a threatened species population. We compared condition indices among three seed-eating bird species–one endangered (Gouldian finch) and two non-threatened (long-tailed finch and double-barred finch)—from two large areas (> 2,830 km^2^) with initial contrasting fire regimes (‘extreme’: frequent, extensive, intense fire; versus ‘benign’: less frequent, smaller, lower intensity fires). Populations of all three species living with the extreme fire regime had condition indices that differed from their counterparts living with the benign fire regime, including higher haematocrit levels in some seasons (suggesting higher levels of activity required to find food), different seasonal haematocrit profiles, higher fat scores in the early wet season (suggesting greater food uncertainty), and then lower muscle scores later in the wet season (suggesting prolonged food deprivation). Gouldian finches also showed seasonally increasing stress hormone concentrations with the extreme fire regime. Cumulatively, these patterns indicated greater nutritional stress over many months for seed-eating birds exposed to extreme fire regimes. We tested these relationships by monitoring finch condition over the following years, as AWC implemented fire management to produce the ‘benign’ fire regime throughout the property. The condition indices of finch populations originally living with the extreme fire regime shifted to resemble those of their counterparts living with the benign fire regime. This research supports the hypothesis that fire regimes affect food resources for savanna seed-eating birds, with this impact mediated through a range of grass species utilised by the birds over different seasons, and that fire management can effectively moderate that impact. This work provides a rare example of applied research supporting the recovery of a population of a threatened species.

## Introduction

Declines in biodiversity in the tropical savannas of Australia are provoking serious policy and management concern [1, 2, 3]. The region is globally significant, having very low rates of land-clearing and other gross physical change [2, 4]. Within Australia, the conservation value of the tropical savannas relative to other regions is also nationally significant; until recently, the tropical savannas had not yet experienced the same extent of biodiversity loss that has affected most of the rest of the continent since European colonisation [5, 6]. The recent realisation that species and ecosystem function can be lost from vast uncleared landscapes with low human population densities, including large protected areas [5, 7], contrasts with the usual global trend [8, 9] and highlights the need for a deeper understanding of how contemporary but less acute threats (changed fire regimes and invasive species) are so profoundly affecting native ecosystems. Further, there is a pressing need to translate this understanding into management responses that will maintain the condition of savanna ecosystems longer-term, including recovering species that have declined [10, 11].

Of the major threatening processes driving biodiversity declines in savannas across the world, changed fire regimes, often with other processes overlaid, have attracted much attention. This is partly due to the integral role of fire in shaping savanna ecology and also its potency and wide use as a management tool [12–17]. Describing how fire regimes have changed over time and understanding the effects of fire on biodiversity have therefore been focal areas for research.

In most of the world’s tropical savannas, increasing human populations and stock densities have led to reduced fire frequencies [18], sometimes precipitating vegetation change that further limits fire spread [19, 20]. In contrast, decreased fire frequency typifies only a relatively small portion of the Australian savannas, mostly in areas with high stock densities (and thus reduced fuel loads) [21]. Across most of the Australian tropical savannas, fires have increased in size, intensity and frequency in recent times, mainly because of a shift in the average timing of fires to later in the dry season (when fuel and weather conditions means that fires burn more intensely and extensively). Prior to European settlement of Australia, Aboriginal people managed fire intensively for thousands of years [22], with numerous, mostly small, low intensity fires, lit for a variety of purposes [23, 24]. These actions tended to reduce the spread and consequence of fires later in the dry season. Following pastoral settlement of the savannas and the widespread displacement of Aboriginal people from their traditional lands (mid 1800s to mid-1900s) Aboriginal fire management was diminished, and fire regimes shifted towards larger and more intense fires, lit by people and lightning later in the dry season, resulting in a higher proportion of the landscape burnt every year [25–27].

A growing body of work, especially from southern Africa and Australia, illustrates the relationships between fire regime changes and biodiversity declines in savannas [13, 15, 16]. However, it has proven surprisingly hard to translate this knowledge into management guidelines for species’ recovery in the Australian setting. Partly, this is because fire ecology is complex and the relationships between fire regimes and biodiversity are both context-dependent and can take a long time to play out. In addition, most fire management planning in Australia is framed around tolerance thresholds of plants and plant communities, rather than those of sensitive fauna species that inhabit those communities [28]. Third, fire is now interacting (in variable, and poorly-described ways) with other novel threats to savannas (introduced herbivores, predators and plants, and climate change) that further complicate impacts [29, 30]. Fourth, research has tended to focus on the consequences of fire on population persistence and abundance rather than the mechanisms underlying declines [28, 31, 32], which can leave the detail of management objectives murky (see [30, 33, 34] for exceptions). Finally, and arguably most importantly, the implications of research results are rarely scaled up into applied management [11, 35–37] (but see [38, 39] for exceptions).

Reviews of studies of fire impacts on various taxa conclude that changes to fire regimes can cause community rearrangement, a range of population-level responses, and declines in taxa with narrower tolerances [29, 35, 40, 41]. The contemporary shift in Australian savannas towards an extreme of frequent large, high intensity fires is thus implicated in the current declines of several groups, including mammals [5], frugivorous, seed-eating, ground-dwelling and riparian birds [29, 42–44], obligate-seeding plants [45] and fire sensitive vegetation [46, 47].

Almost half of the seed-eating bird species in Australia’s tropical savannas have declined since European settlement [44]. The declines are most pronounced in areas with relatively greater changes in fire regimes and grazing [48, 49]; both factors change the species composition of the grass layer and can reduce the seed output of individual plants [50–52]. All grass seed production in the tropical savannas occurs in the three to four months of the wet season, with particular grass species flowering and seeding in a moderately predictable sequence for any ambient rainfall and fire pattern [53]. At the end of each wet season, seeds from some annual grasses are produced in large quantities, and these seeds (mostly shed to lie of the ground surface) form a staple for grass seed-eaters over the dry season months. Once the next wet season begins, the annual seeds germinate, and seed-eaters move on to other (mostly perennial) grass species that flower and then set seed over the course of the wet season [50, 53–55]. Seed-eating birds may move across the landscape to take advantage of staggered seeding events, caused by the spatio-temporal intersect between fire and rain events [55, 56]. However, gaps in any part of this seeding sequence–either because key grass species (or their seed production) drop out of the system, or because fire patterns take on a scale larger than the movement capabilities of the birds–will presumably cause food resource shortages and hence population decline. Earlier authors have suggested that the early wet season is the most likely period for such a gap, when the dietary shift between previously abundant annual grass seeds (that germinate with the rain) and perennials (which grow with the rain) takes place [53–57].

The grass-seed eating Gouldian finch *Erythrura gouldiae* has exhibited particularly marked declines, with extreme contractions in distribution and population size over the past 40 or so years [58, 59]. It has been the subject of a relatively large research effort since the 1990s, sparked by concern that the declines signalled widespread dysfunction in the savanna grass layer, and that this heralded an impending biodiversity crisis across northern Australia. This research proposed that Gouldians may be especially susceptible to changes in the grass layer because they have a narrower diet than other seed-eaters [57], and because they are obligate hollow-nesters [60, 61] which curtails their usual dispersive movements and limits foraging (during breeding) to an area around nesting sites. Other sympatric finch species have both a broader diet, and are not obligate hollow-nesters. However, despite these insights, we are still unable to precisely link fire regime changes to food resource bottlenecks in a way that informs restorative fire management design, because these hypotheses have not been tested in an applied management framework [49, 55].

Here, we report on a landscape-scale applied management experiment that aimed to understand the relationship between fire and a key guild (grass seed-eating birds) in Australia’s tropical savannas, in order to design management for species’ recovery. Three co-occurring species of seed-eating finches–the Gouldian finch (nationally Endangered), long-tailed finch *Poephila acuticauda* and double-barred finch *Taeniopygia bichenovii* (both non-declining species)–all endemic to the tropical savannas of northern Australia, were considered in this study. We examined their condition in two large adjacent areas subjected to contrasting fire regimes (a northern area with an ambient regime of frequent, extensive and intense fires; a southern area with infrequent, small and low intensity fires). Both areas were on Mornington Wildlife Sanctuary, which is owned by the Australian Wildlife Conservancy (AWC). Following acquisition, AWC sought to improve the condition of savanna habitats by managing fire at progressively larger scales [39]. Thus, over the latter years of the eight-year study, the fire regime at the northern study site was shifted until it matched the relatively benign regime of the southern area. During this time, we tested the finch population response to this fire regime change. Typical response parameters such as population size, individual survival and reproductive success are impractical to assess in mobile and (in the case of Gouldian finch) rare species. Instead, we measured condition indices across populations and fire treatments, with the assumption that individuals in populations experiencing environmental stress will be in poorer condition. This approach has been successfully used in analogous studies that aimed to identify environmental stressors on populations (reviewed in [62, 63]). Further, a recent survey of condition measures in a range of north Australian finch species successfully discriminated declining from non-declining species, with condition in declining species being poorer in areas subjected to frequent, intense fires, or heavy grazing [64, 65].

In this study, we predicted that the initial survey should show that the condition indices of Gouldian finch populations (but not long-tailed or double-barred finch populations) were sensitive to the existing contrast in fire regime between northern and southern areas. Based on earlier research [53–57], we expected to see this condition divergence most clearly in the early wet season. As the fire regime in the northern study area was manipulated to resemble that of the southern area, we expected to see any condition differences between north and south diminish. Our objective was to discover if fire management could be applied to promote recovery in a threatened savanna bird species.

## Methods

### Study site

The study was carried out at Mornington Wildlife Sanctuary in the central Kimberley, north-west Australia (17°30 S; 126°06 E). This 313,000 ha property is owned and managed by AWC, a private, non-profit conservation organisation. Prior to its acquisition by AWC in 2001, Mornington had been operated as a cattle station since the 1920s, with an extensive grazing system and a free-ranging herd of 6,000–8,000 head, plus a similar number of feral donkeys and horses. The property is dominated by open savanna eucalyptus woodlands with a hummock grass (*Triodia* spp.) and/or tussock grass understorey. The research was carried out in two areas of the property: a northern ‘Zone’ and a southern ‘Zone’; the centres of these Zones were 80 km apart.

### Rainfall patterns

The climate of the study area is strongly monsoonal, with over 80% of the annual rainfall falling between December and March each year. Rainfall can influence the quantity and timing of seed production [66]. To investigate the possibility of a difference in rainfall between northern and southern zones, and/or a systematic change in rainfall over time, we calculated rainfall statistics (annual rainfall, rainfall in the lead-up to the wet season (Sept-Dec), and the cumulative monthly rainfall pattern through the year) from July one year to June of the next year for 2004–05, 2005–06,…2010–11. This 12-month period captures each monsoon season, and also matches our successive finch sampling periods (September-April, see below). Rainfall data were collected by AWC staff from manual and automatic weather stations in both the northern and southern zones.

For the purposes of this study, we identify four distinct seasons through the year that are relevant to the feeding ecology of the finches (see below): dry season (May-August; cooler temperatures, negligible rain); late dry season (September-December; high temperatures, isolated storms); early wet season (December-January; high temperatures, accumulated rainfall rising to c. 200 mm); wet season (January-March; variable temperatures, high rainfall). The dates of transitions between each season vary between years, by up to a month, and the annual rainfall can vary substantially between years, with consequences for the extent of grass growth and therefore fire [66].

### Fire regimes

We described fire regimes in the north and south Zones of Mornington using archived MODIS satellite imagery, downloaded from the North Australia Fire Information website (www.firenorth.org/nafi2). Using Arcview 9.3, we centred a circle of 60 km diameter (i.e with area 2,830 km^2^, and with 20 km separating the external boundaries of the Zones at their closest point, over each of the northern and southern Zones, and intersected this with the annual fire scar data to calculate the % of the Zones that were unburnt in the year leading up to sampling, the % unburnt for two years prior to sampling, and the % unburnt for three years (or more) prior to sampling.

At the beginning of the study, the northern and southern Zones of Mornington had contrasting fire regimes. The north experienced more frequent, larger and more intense fires compared to the south of the property, leading to smaller extents of vegetation that had not been burnt for two or more years. (Thus, the northern Mornington had fire regimes closer to the contemporary patterns occurring over large parts of northern Australia). This north-south difference arose because topographical features (major rivers, fringing sandstone ranges) on the southern boundary of Mornington act as natural firebreaks to uncontrolled fires from that direction. The north, however, is vulnerable to uncontrolled fires from all points of the compass. Between 2006 and 2012, AWC used prescribed burning to progressively reduce the frequency, size and intensity of fires in the north until the fire regimes and spatial metrics were similar to those in the south [39].

### Relevant species ecology

The three finch species are all grass seed specialists. During the dry season (April-December), seed of native sorghum (*Sorghum stipoideum*) forms the bulk of the diet in the region, especially for Gouldian finches and long-tailed finches. The store of sorghum on the ground declines (from seed predation, fire, and as the seeds self-drill into the ground) steeply throughout the dry season [67] with surviving seeds germinating when the wet season begins (c. December) [52]. Sorghum seed is no longer available as a food source [50, 55] after cumulative rainfall of about 50 mm [66]. The same rains stimulate the growth of small annual grasses (e.g. *Urochloa holosericea*) and perennial grasses that provide seed for finches during the wet season (in this study region, especially *Alloteropsis semialata*, *Triodia* spp., *Xerochloa laniflora*, *Chrysopogon fallax*, *Whitechloa biciliata*, and also smaller amounts of *Aristida* spp., *Brachiara* spp., *Eriachne* spp., *Eragrostis* spp.). At our study site, early-seeding spinifexes (*Triodia* spp.), which often produce flowers and seeds ahead of other grasses [53] (Legge, unpublished data), also help to transition finches from the dry season sorghum stores to wet season grasses. Gouldian finches specialise more exclusively on grass seed compared to other finch species that broaden their diet in the late dry and wet seasons to include other plant materials (e.g. the fleshy coverings, or arils, of acacia seeds) and invertebrates [57] (Legge, unpublished data) if seed becomes scarce [68]. By the end of the wet season, however, sorghum again produces seed in copious quantities, and finches switch back to this as their main food source [55].

Breeding is concentrated from February to July [60, 69] but can extend beyond these months, especially for long-tailed and double-barred finches. Gouldian finches are obligate tree-hollow nesters, long-tailed finches nest mostly in tree hollows but will also build nests in other structures (mistletoe clusters, grass tussocks, etc), and double-barred finches build domed nests in shrubs and other low vegetation [61, 70, 71].

Moult is most active in September to November for all three finch species, but especially so for Gouldian finches which tend to carry out a full moult over a shorter period [60]. All three species are short-lived, but particularly Gouldian finches, which usually live for only 1–2 years [72].

While Gouldian finches can move longer distances than the other two species [72], and could potentially move between the northern and southern sampling zones, our banding records indicate that this occurred either very rarely, or not at all. Of 719 individuals that we colour-banded, we resighted 19% on 359 occasions. Of 1355 birds that we banded with uniquely numbered metal bands, we recaptured 67 individuals (5%). All resights and recaptures occurred in the zone in which the bird was originally banded; we did not detect any movement between the zones. Resights and recaptures mostly occurred within one year of the original capture, but the longest gap between banding and recapture was 5 years, and this individual had moved only 15 km.

### Study design

The study took place between mid-2004 and mid-2011, and was split into two parts. In the first four years, the north and south of Mornington had contrasting fire regimes that we exploited to examine whether condition measures for the bird species varied between the two areas subject to contrasting fire regimes. Having identified a correlation between condition and fire, especially for Gouldian finches, we predicted that the condition indices of the northern populations should converge on those of the south as the northern fire regimes were actively managed over the following years to resemble those of the south.

For both the first and second parts of the study, we sampled Gouldian, long-tailed and double-barred finches in the northern and southern zones of the property in three seasons (late dry season, early wet, wet season) between late September and April each year; this period encompassed the key seasonal diet shifts as well as major life history events (moulting, breeding) that can be energetically expensive. Preliminary analyses of condition measures collected throughout the calendar year confirmed that this period was the most dynamic.

We captured finches using mist nets and walk-in traps set at waterholes and feeding sites, sometimes using playback of calls to attract over-flying birds. All birds were individually banded. To assess condition, we measured fat stores, muscle status, haematocrit, and plasma corticosterone. We scored the amount of fat in the furculum (the area below the bird’s throat, between the clavicles) as 0 = no fat; 1 = thin strip of fat; 2 = half-filled with fat; 3 = fat flush with surface of throat. We also assessed the contour of the pectoral muscles around the keel (as 0 = emaciated; 1 = protruding keel; 2 = keel visible but muscles well-developed; 3 = keel not protruding at all from the surrounding plump muscle). Keel score indicates energy and protein reserves, and fat stores indicate energy reserves [62, 63].

For every captured bird, a blood sample of 50–70 μl was collected from the brachial vein into a heparinised haematocrit tube, and stored at 4°C until it could be processed (usually within four hours of collection). We spun the blood samples in a Hettich Haematokrit 210 centrifuge for 7 minutes at 16,060 g, then measured the percentage of packed cell volume with a haematocrit reader. Haematocrit is a non-specific indicator of general health and activity; reduced haematocrit can indicate poor nutrition, high parasite loads, disease, and/or increased activity levels [73].

Finally, we measured the plasma corticosterone (CORT) concentration of a subset of 121 Gouldian finches sampled during 2004 to 2006, from the northern (n = 56) and southern (n = 65) zones. Chronically high levels of CORT, the primary avian glucocorticoid, can result in reduced growth and reproduction, immunosuppression, neuronal cell death, severe protein loss and mortality [73–75]. Plasma was withdrawn from the spun-down haematocrit tube using a fixed-tip syringe, transferred to a plastic cryovial, then frozen at -80°C in liquid nitrogen. Occasionally we stored samples at –20°C in a portable freezer temporarily, until we had access to the nitrogen cylinder. Total plasma CORT was measured from 5μL aliquots of whole plasma from each bird using an EIA Kit ACE^TM^ Competitive Enzyme Immunoassay for corticosterone (Cayman Chemical Co. Ann Arbor, Michigan, USA). This assay uses competitive binding between CORT contained in the plasma aliquot and a fixed amount of a corticosterone-acetylcholinesterase (AChE) conjugate for a limited number of antiserum binding sites in the well of the assay plate. The antiserum-CORT complex is bound to an immunoglobulin and combined with a reagent that contains the substrate AChE to produce an enzymatic reaction with a yellow colour that can be read by a spectrometer [76]. Two standard CORT solutions (one high concentration and one low) were tested along with finch plasma on all plates; both the intra-assay and inter-assay variability was < 5%.

### Analysis

#### 1. Condition variation among populations of three species, pre-management

We used a linear regression modelling approach in Genstat 8 to examine the influence of Season (late dry, early wet, mid wet) and Zone (north; south) on the condition measures of keel score, fat score, haematocrit, and CORT (Gouldian finches only). We examined all two-way interactions as well as the single three-way interaction (between Species, Season, and Zone), pooling data from the years 2004–05, 2005–06 and 2006–07 for this analysis. We were particularly interested in how Zone (including its interactions with other variables), as a surrogate for fire regime, affected the condition indices.

When considering the CORT data from the subsample of Gouldian finches, we first needed to account for the effect of handling on CORT response. We regressed CORT concentrations of each sample against the time interval between capture and handling of that bird. All birds in this sample had been bled within one hour of capture. The relationship was significant (F_1,119_ = 8.66; p = 0.004). We used the residuals, which represent the release of CORT in response to handling relative to the overall mean response, and used these in further analyses of the influence of various factors on CORT.

#### 2. Condition variation in response to applied fire management

Based on the results of the initial condition survey (i.e. analysis 1 above), we restricted this analysis to seasonal haematocrit, keel score in the mid wet season, and fat score in the early wet season, as these measures differentiated northern populations (and especially northern Gouldian finches) from their southern counterparts (i.e. they were potentially related to fire regime). Again using Genstat 8, we examined how Management (pre and post fire management intervention), Zone and Season (for haematocrit) were related to the condition indices. We analysed each species separately. We predicted that the condition indices of northern populations should become more similar to those of the south when the fire regime in the north was managed to resemble that of the south. For this analysis, we grouped years 2004–05 to 2007–08 as pre-management, and 2008–09 to 2010–11 as post-management, based on inspection of when fire regime metrics (such as the extent of unburnt vegetation) changed (i.e. from 2008 on; see Results). Finally, to cross-check the relationship between fire management and condition, we plotted the haematocrit for the northern population of Gouldian finches against the percentage of 2+ year unburnt vegetation.

### Permits and approvals

The study was carried out by the Australian Wildlife Conservancy on a pastoral lease it owns and manages. To catch and handle wildlife, we obtained approvals from Western Australia’s Department of Parks and Wildlife (Regulation 17 Licence to Interfere with Wildlife for Scientific Research from WA DPaW; Regulation 23 Licence to Take and Mark Fauna for Research Purposes from WA DPaW), as well as a Licence to Use Animals from the Department of Local Government, Western Australia. The Western Australian Department of Parks and Wildlife (DPaW) Animal Ethics Committee reviewed and approved all aspects of the capture, handling and experimental design of this study (permit numbers: CAEC 6/2005; DEC AEC 43/2007; DEC AEC 2010/35). To capture birds in mistnets and band them (details of field methods are described above), SL obtained a Banding Authority and an Approved Project (which sets out the methods of the study and the number of birds to be captured) from the Australian Bird and Bat Banding Authority. The study design and imposed management were consistent with research and management priorities established for the conservation of the Gouldian finch in its national recovery plan [59]. All permits were reported on, reviewed and renewed annually.

## Results

### Rainfall patterns

**Fig 1:**
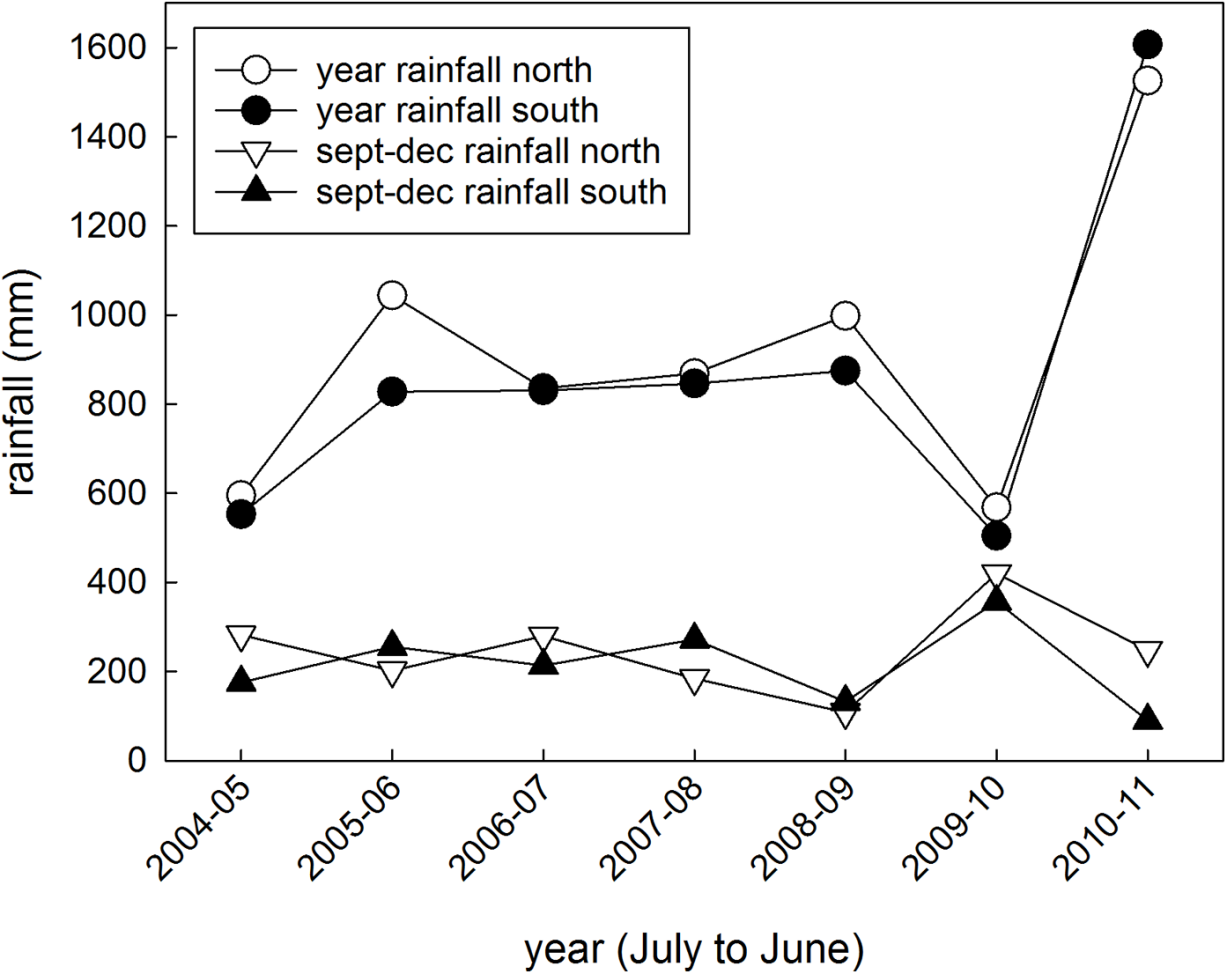
Annual and pre-wet season rainfall statistics. Yearly rainfall (July to June) and in the lead-up (Sept to Dec) to the wet season, for the northern and southern zones of Mornington.

**Fig 2:**
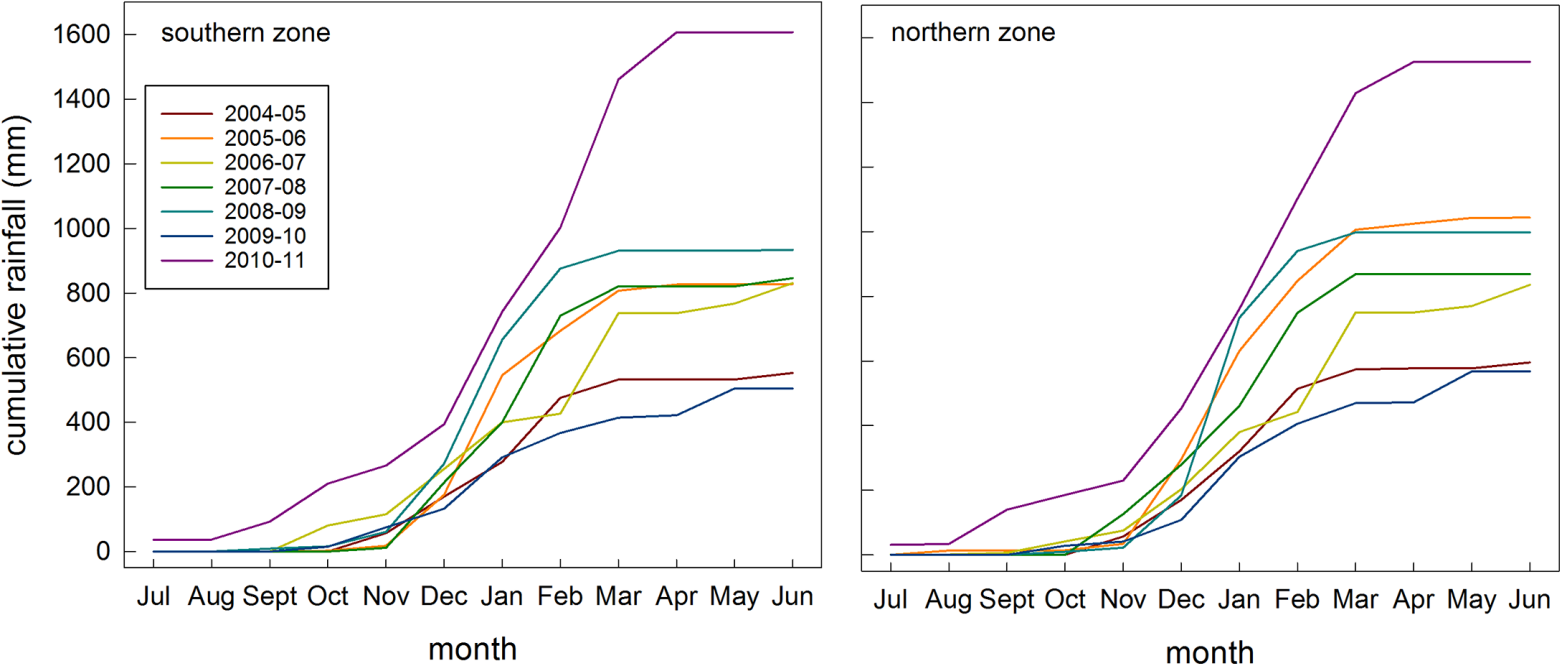
Cumulative rainfall statistics. Cumulative rainfall during July to June each year, for the northern (right) and southern (left) zones in the study area.

The mean annual rainfall from July to June over the period 2001–13 was similar in the south (828 mm) and north (862 mm). The year to year totals between 2004 and 2011 for northern and southern were also alike (Fig 1), as was the rainfall in the lead-up to the wet season (Sept-Dec; south: 211 mm; north: 231 mm; Fig 1). The pattern of cumulative monthly rainfall was similar between the zones (Fig 2), with each experiencing very high rainfall during 2010–11, an unusual dry spell in February 2007, and a similar wet to driest year ranking that did not correspond to a systematic pattern (i.e. 2010–11 >> 2005–06 and 2008–09 and 2007–08 and 2006–07 >> 2004–05 and 2009–10).

### Fire regimes

**Fig 3:**
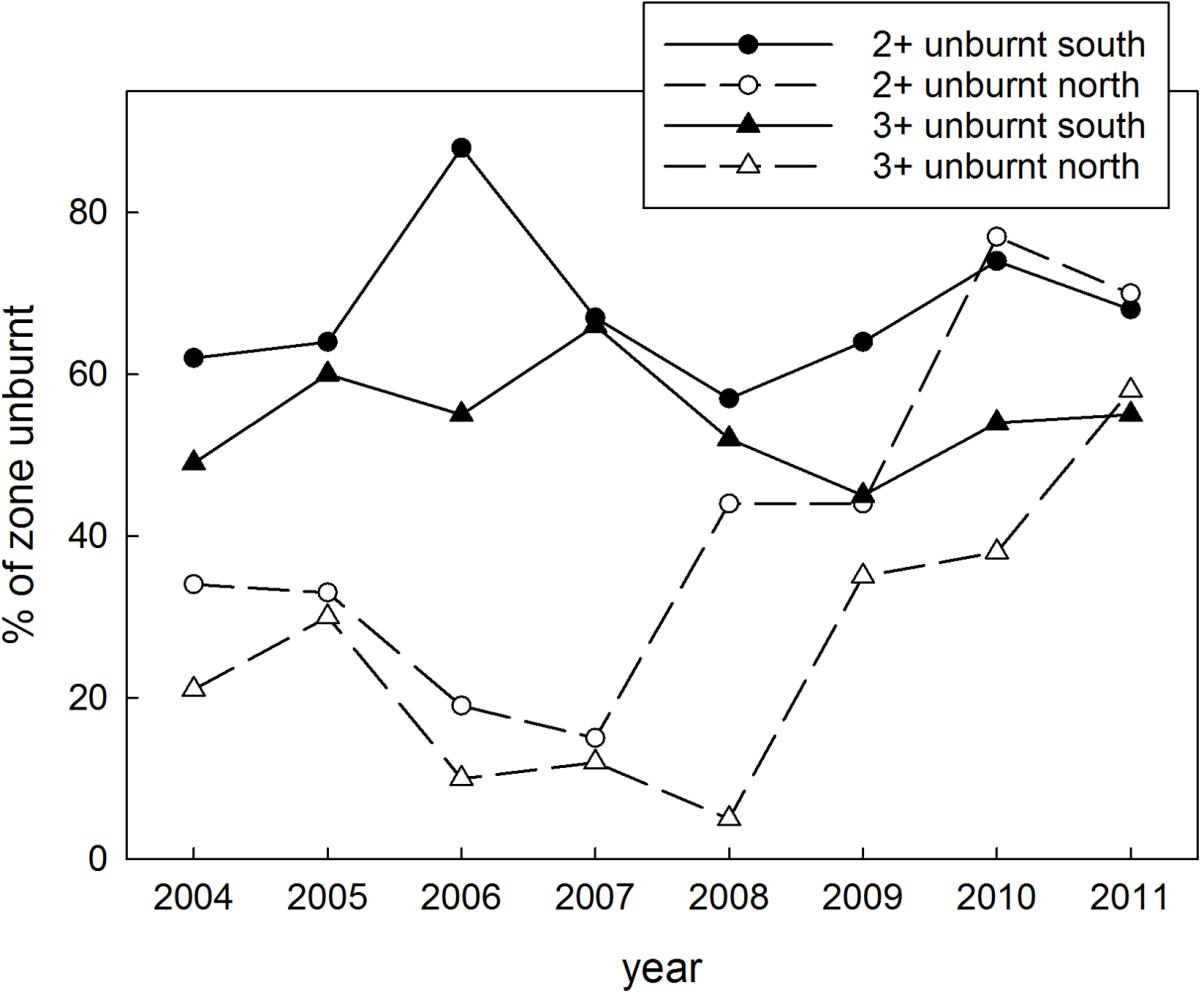
Fire regime metrics. The percentage of the northern and southern zones with vegetation of two, or three plus years of age post-fire, during the years 2004–11.

**Fig 4:**
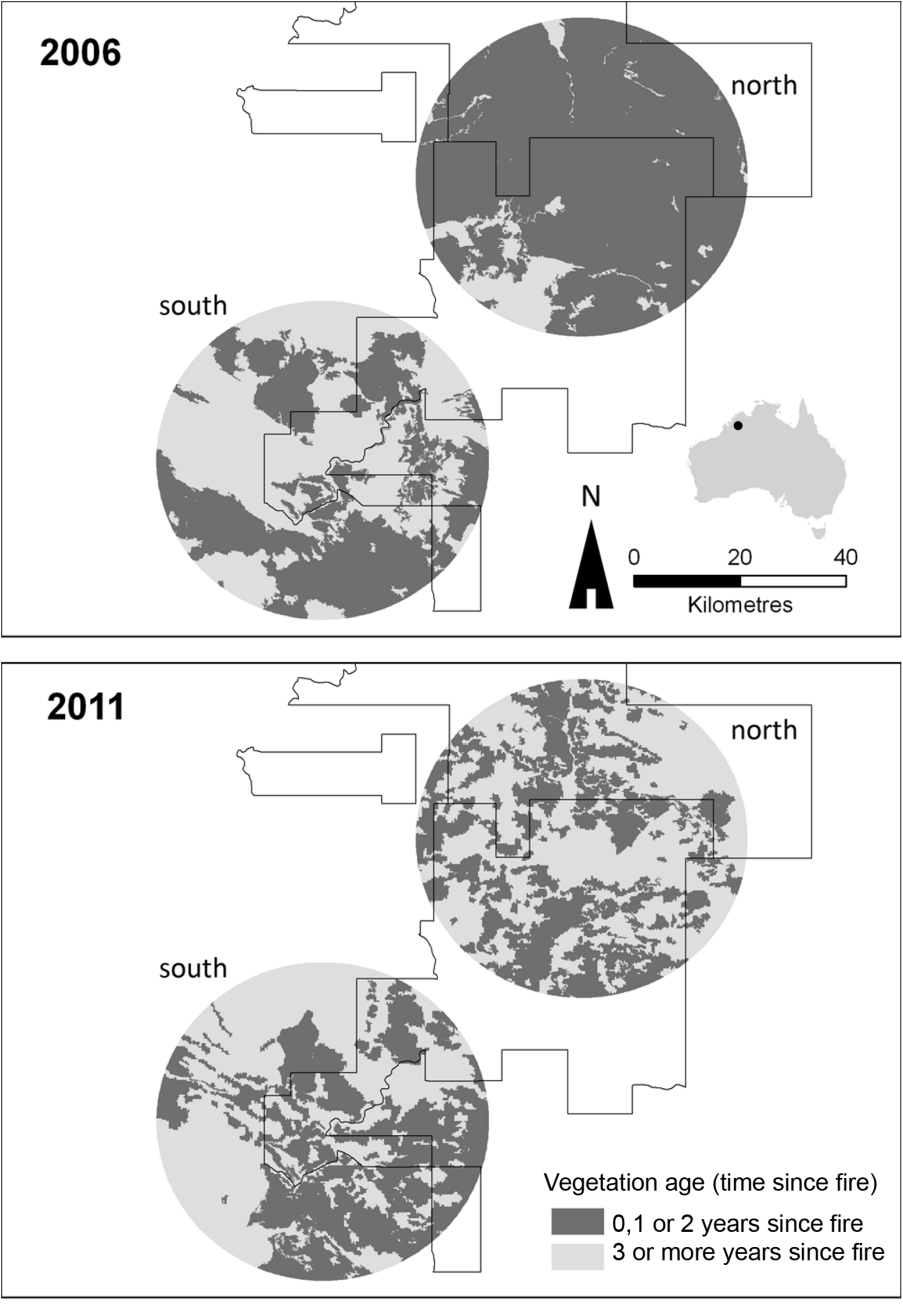
Fire regime metrics. The spatial dispersion of long-unburnt (i.e. 3 or more years since fire) vegetation at the end of 2006, and the end of 2011. The inset shows the location of AWC’s Mornington Wildlife Sanctuary.

In the first half of the study, the southern zone was characterised by smaller and less frequent fires compared to the northern zone, leading to greater areas of vegetation that had not been burnt for two or more, and three or more years, distributed more evenly across the zone (Figs 3 and 4). From 2008 on, in response to imposed management, the fire regime in the north became increasingly similar to that of the south, so that by 2011, the extent (and spatial arrangement) of vegetation not affected by fire for two and three or more years were similar between the zones (Figs 3 and 4).

### Sample of finches

Over the eight years of the study we collected data on condition for 1524 long-tailed finches, 1094 Gouldian finches, and 1012 double-barred finches. Individuals were only rarely resampled: 8%, 5%, and 14% of long-tailed, Gouldian and double-barred data respectively were from individuals recaptured in subsequent seasons or years. We discarded a small number of keel and fat scores that had been incorrectly recorded (e.g. given a score outside the standard range); we discarded haematocrit measures from blood samples that were too small to be reliably processed, lysed before processing, or spun out in the centrifuge.

#### 1. Condition variation among populations of three species, pre-management

**Fig 5:**
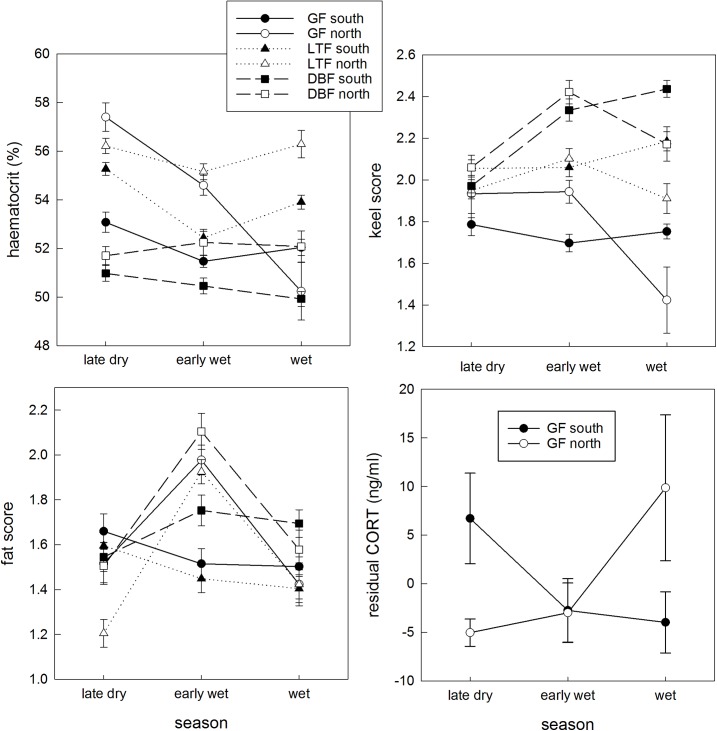
Seasonal condition indices of finch populations. a) Haematocrit; b) Keel score; c) Fat score of Gouldian (GF), long-tailed (LTF) and double-barred finches (DBF); and d) residual CORT concentration for Gouldian finches, from the northern and southern zones of the property with contrasting fire regimes in the early years of the study. Data are means with standard errors. Statistics and sample sizes provided in Table 1.

Significant predictors of the variation in keel score, fat score, haematocrit and residual CORT are described in Table 1 and Fig 5.

**Table 1:** Summary of the analyses of condition indices for three finch species living in zones with contrasting fire regimes, pre-management.

		Haematocrit		Keel score		Fat score		Residual CORT	
		n = 2009		n = 2179		n = 2179		n = 121 (GF)	
	df	F	p	F	p	F	p	F	p
Zone.Species.Season	4	**7.11**	**<0.001**	0.46	0.765	1.99	0.093	n/a	
Species.Season	4	**12.23**	**<0.001**	**12.8**	**< 0.001**	**3.2**	**0.013**	n/a	
Zone.Season	2	2.54	0.079	**13.1**	**<0.001**	**34.4**	**<0.001**	**5.48**	**0.005**
Zone.Species	2	**8.95**	**<0.001**	**5.33**	**0.005**	1.09	0.337	n/a	
Season	2	**29.1**	**<0.001**	**3.43**	**0.032**	**12.7**	**<0.001**	**1.67**	**0.194**
Species	2	**88.3**	**<0.001**	**128.1**	**< 0.001**	**10.9**	**<0.001**	n/a	
Zone	1	**34.7**	**<0.001**	0.75	0.385	2.32	0.128	0.013	0.909

Model statistics for variation in haematocrit, keel score and fat score, for Gouldian, long-tailed and double-barred finches in relation to Species, Zone and Season; and for residual CORT concentration for Gouldian finches; in the early years of the study before fire regimes were changed. Sample size for each analysis is shown in the relevant column.

Haematocrit: Northern populations of all species had higher haematocrits than their southern counterparts in each seasonal sampling period (except for northern Gouldian finches in the wet season, because of a large seasonal decrease in haematocrit for this population). The three-way interaction between Species, Zone and Season was significant, with northern Gouldian finches showing a markedly different seasonal pattern to all other populations, losing 7.4% of haematocrit between the late dry and wet seasons (Fig 5). Southern Gouldian finches also lost haematocrit from the late dry to the wet season, but to a much smaller extent (1.7% drop). Long-tailed finches in the south lost 3% haematocrit between the late dry and the early wet season, but then reverted to nearer original levels later in the wet season. All other populations fluctuated less than 1% (up or down) from their late dry season average through the following two seasons.

Keel: The keel scores of southern populations were stable or increased from the late dry to the wet season, but the keel scores of northern populations were stable or decreasing over time. Double-barred finches had higher keel scores as the seasons progressed, but the scores for long-tailed finches remained similar over time, and the keel scores for Gouldian finches decreased. The keel score decrease for northern Gouldian finches was especially marked, although the interaction between Species, Zone and Season was not quite significant (p = 0.07; Table 1; Fig 5).

Fat: Northern populations had similar or less fat than their southern counterparts in the late dry season, but the northern fat scores increased strongly to be much greater than southern populations in the early wet season (Table 1; Fig 5).

CORT: Residual CORT in Gouldian finches in the northern population increased from the late dry through to the wet season, whereas residual CORT in the southern population decreased over these seasons; Fig 5).

Overall, the condition indices that differentiated northern from southern populations were (a) seasonal haematocrit pattern (markedly different for northern Gouldian finches compared with all other populations), (b) the keel score in the wet (reduced for northern populations, especially so for Gouldian finches), and (c) fat score (higher in the early wet for the northern populations of all three species). We focus on these three specific measures in the next analysis, which examines variation in these condition indices as the fire regime in the northern zone was changed. Although residual CORT in Gouldian finches also varied with fire regime, we discontinued our sampling because it was a logistically more difficult measure to obtain during fieldwork in very remote conditions.

#### 2. Condition variation in response to applied fire management

**Fig 6:**
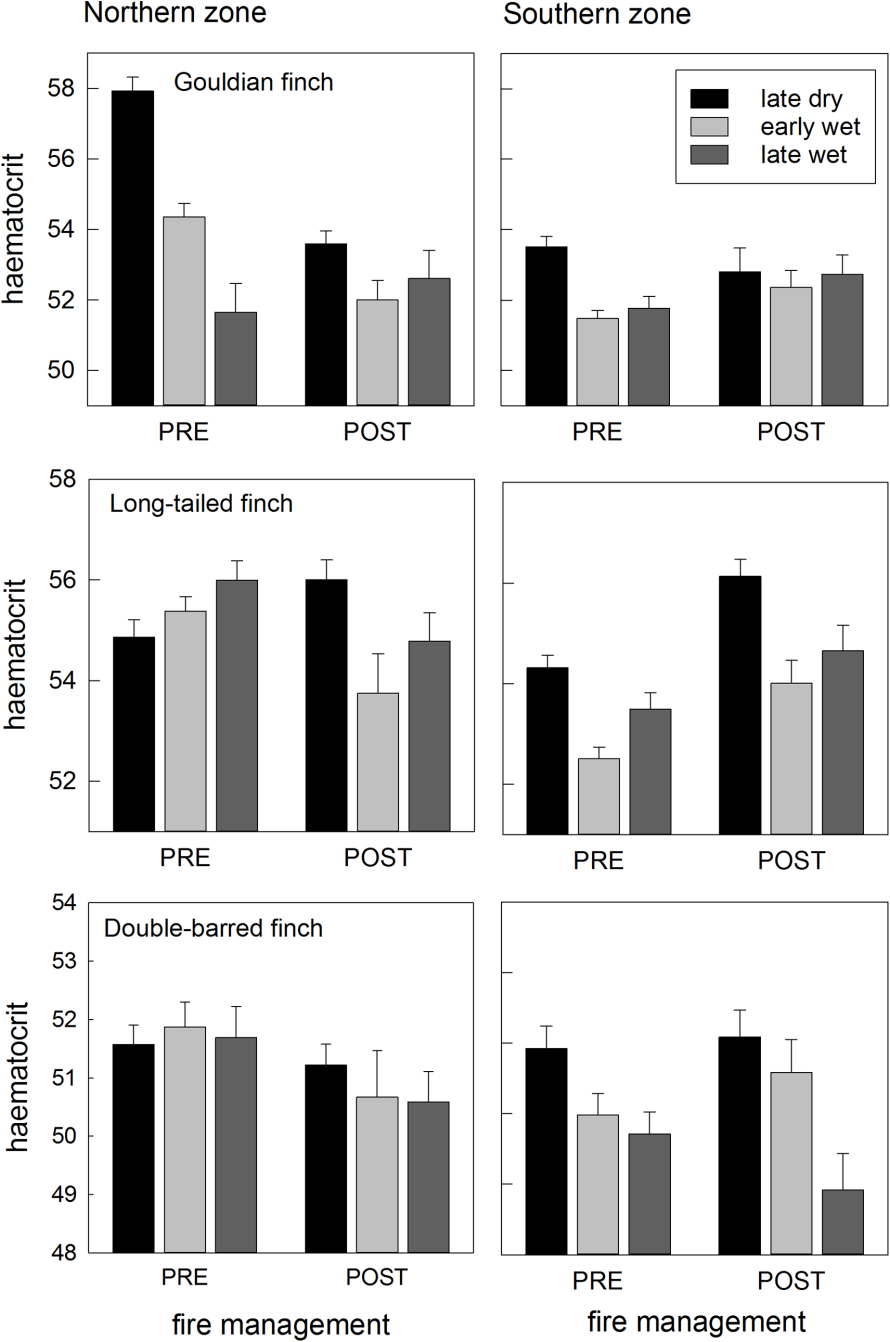
Changes to seasonal haematocrit profiles before and after fire management. Seasonal haematocrit patterns for northern populations of Gouldian, long-tailed and double-barred finches on the left, and each species’ southern population counterpart on the right. Bars are means (with standard errors). The scale of the vertical axis is similar for each north-south species pair, to aid visual comparison within species, but varies among species.

**Fig 7:**
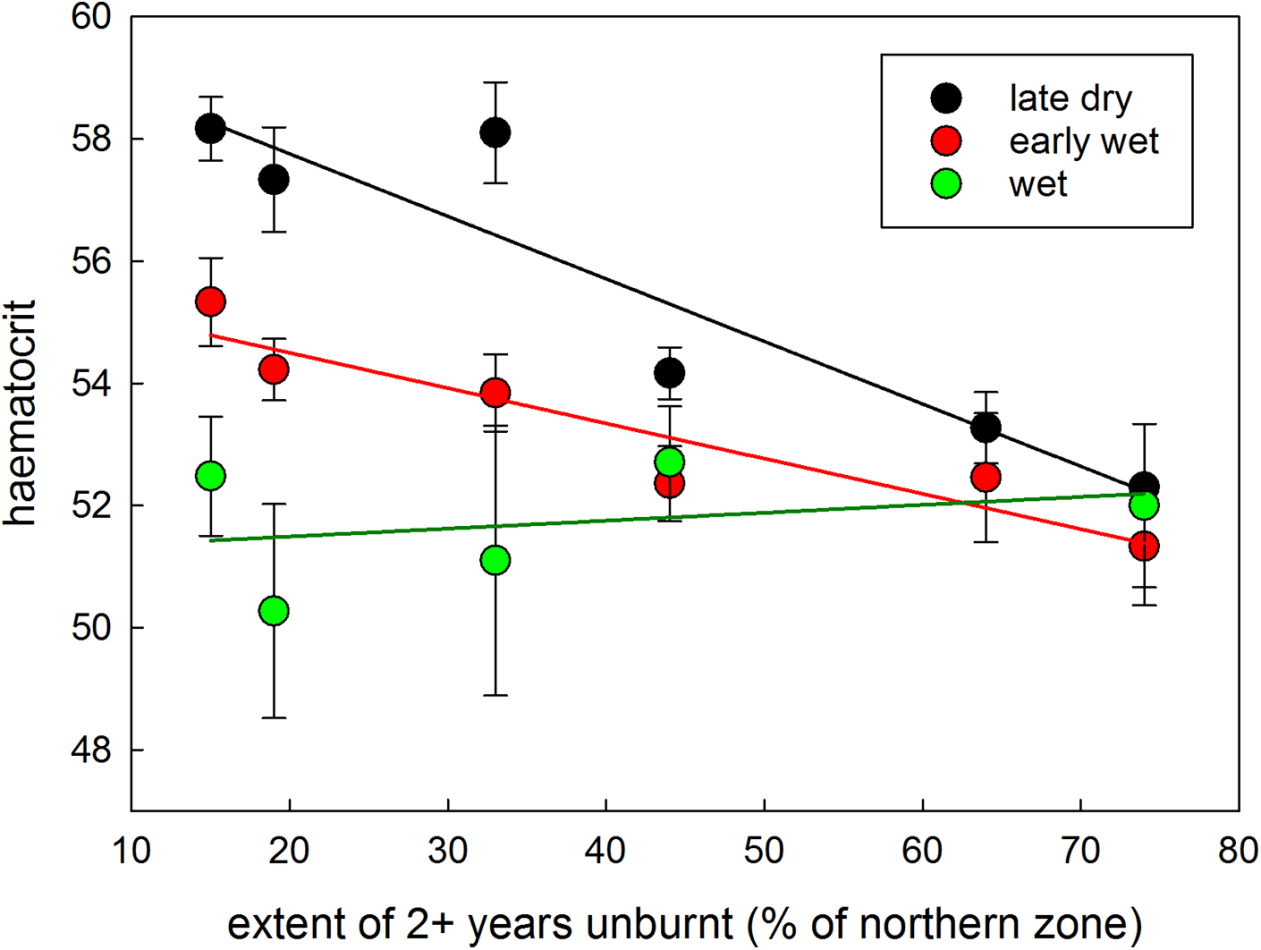
Changes in the haematocrit vales in the late dry, early wet, and wet seasons for northern populations of Gouldian finches as the extent of 2+ year old unburnt vegetation (measured at the end of each year) increases. Dots depict the means (and standard errors) of the data collected from finches from the late dry of that year (i.e. after the dry season fires have taken place), through to the wet of the following year (there are no substantial fires during the wet season); to aid illustration, lines are regressed through the values for each season.

**Fig 8:**
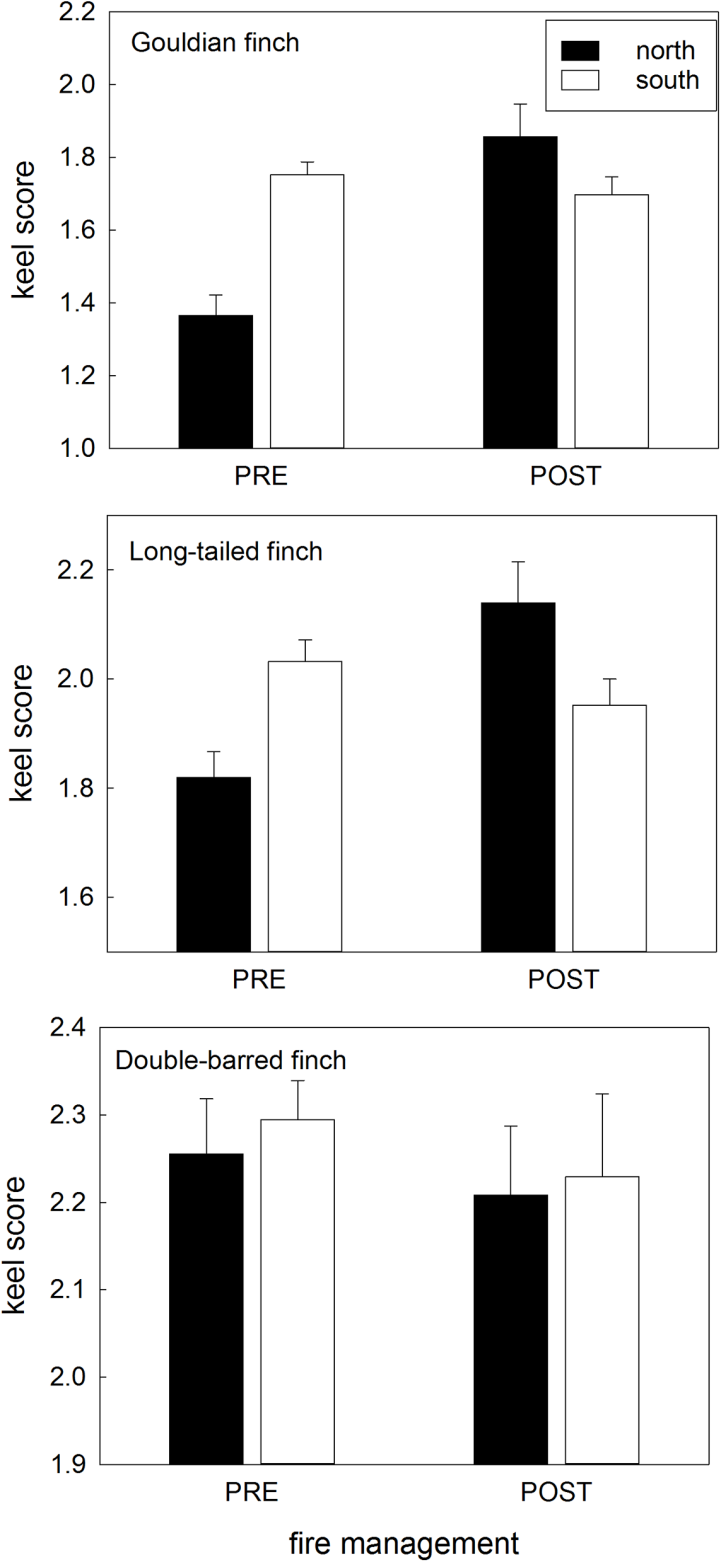
Changes to keel score with fire management. Keel score in the wet season for each species and each population (northern/southern) before and after fire management was imposed. Data are means with standard errors.

**Fig 9:**
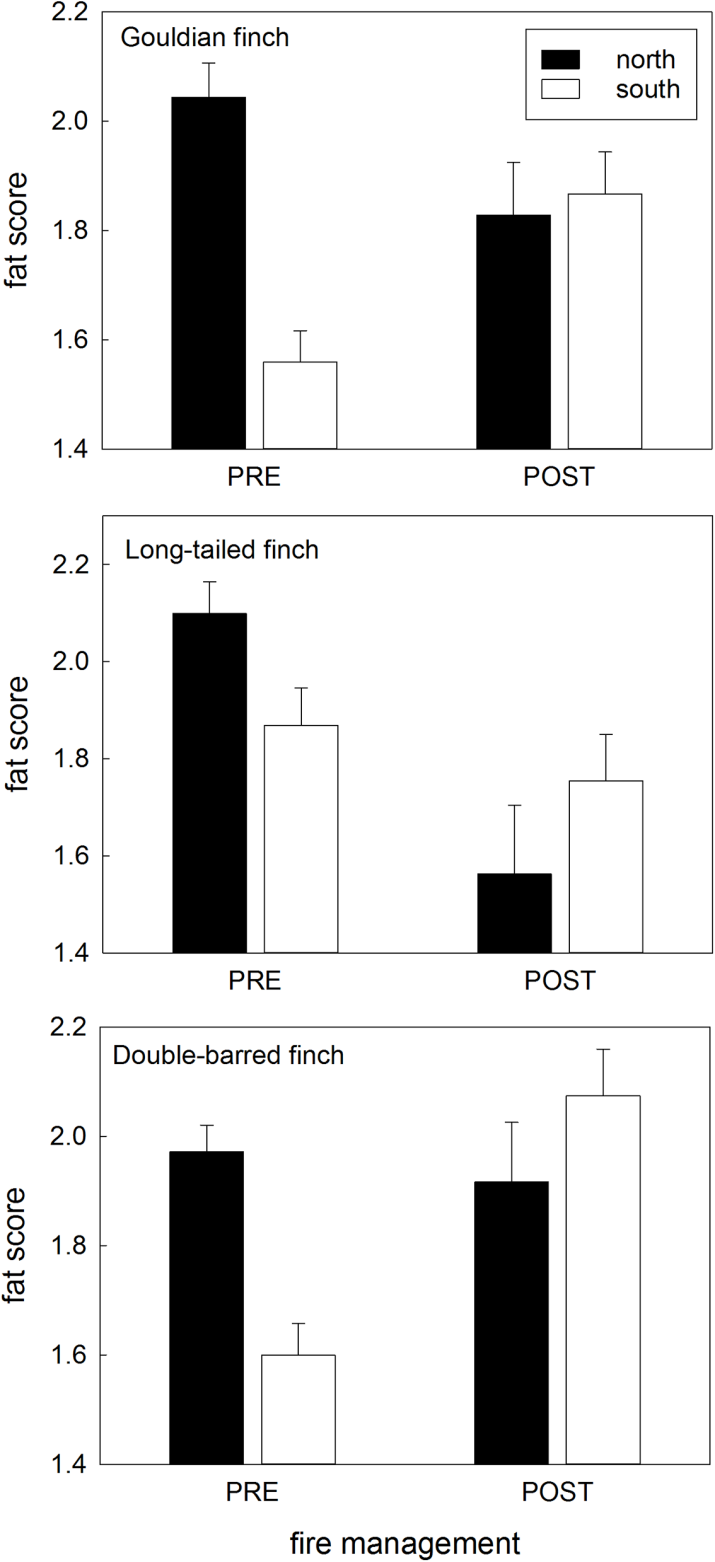
Changes to fat score with fire management. Fat score in the early wet season for each species and each population (northern/southern) before and after fire management was imposed. Data are means with standard errors.

Northern populations experienced changes in each of the three condition indices as fire regimes changed from 2008 on, but southern populations showed little or no such condition variation over the years (Table 2; Figs 5–9).

**Table 2:** Summary of the analyses of condition indices for three finch species, as fire regimes in two zones converged following the imposition of fire management.

		Gouldian		Long-tailed		Double-barred	
	df	n = 1043		n = 1253		n = 837	
**a) Seasonal haematocrit**		F	p	F	p	F	p
Management.Season.Zone	2	**2.87**	**0.050**	2.24	0.107	0.338	0.713
Management.Season	2	**8.12**	**<0.001**	**4.76**	**0.009**	0.794	0.453
Management.Zone	1	**8.38**	**0.004**	0.872	0.351	0.472	0.492
Season.Zone	2	**4.99**	**0.007**	2.50	0.082	2.27	0.104
Management	1	**16.3**	**0.029**	**16.8**	**<0.001**	0.066	0.798
Season	2	**16.2**	**<0.001**	**10.2**	**<0.001**	**4.20**	**0.015**
Zone	1	**17.4**	**<0.001**	0.033	0.859	1.04	0.307
**b) Wet season keel score**							
Zone.Management	1	**18.2**	**<0.001**	**11.3**	**<0.001**	0.012	0.914
Management	1	**11.7**	**<0.001**	**4.06**	**0.045**	0.466	0.495
Zone	1	3.12	0.079	0.041	0.840	0.215	0.643
**c) Early wet season fat score**							
Zone.Management	1	**7.10**	**0.008**	**5.63**	**0.002**	**3.89**	**0.049**
Management	1	0.215	0.643	3.55	0.060	**9.23**	**0.003**
Zone	1	**5.13**	**0.024**	0.93	0.336	0.030	0.862

**Seasonal haematocrit:** The northern population of Gouldian finches experienced a significant change in their seasonal haematocrit profile following the imposition of fire management, changing from a marked seasonal decline before fire management, to no seasonal pattern after fire management, thus matching the pattern of the southern population of Gouldian finches (three-way interaction between Management, Season and Zone significant for Gouldian finches (p = 0.05; Table 2; Fig 6). The northern population of long-tailed finches showed a seasonal increase before fire management, but a seasonal decrease after fire management that resembled the profile of the southern population (p = 0.107; Table 2; Fig 6). In both species, the seasonal haematocrit profiles before and after management in the southern zone were similar, but the seasonal profile in the northern zone differed before and after management (Table 2; Fig 6). Double-barred finches showed a small seasonal decline that was similar between northern and southern zones, and before and after fire management Table 2; Fig 6).

The change in the seasonal haematocrit profile appeared to be linked to the change in fire regimes resulting from management. To cross-check this implication, we plotted the haematocrit for the northern population of Gouldian finches against the percentage of 2+ year unburnt vegetation. Fig 7 shows that as the extent of 2+ year old vegetation increases, the values for haematocrit in the late dry, early wet, and wet seasons all gradually changed (i.e. converged) in a manner consistent with the pattern revealed in Table 2A and Fig 7.

**Keel score:** The wet season keel scores of the northern population of Gouldian finches before fire management were lower than that of the southern population, but increased after fire management to be similar to the southern population keel score (which did not change) (Table 2; Fig 8). The keel score of northern long-tailed finches also increased when fire management was imposed, whereas the keel score of the southern population decreased non-significantly (Table 2; Fig 8). The keel scores of double-barred finches remained similar before and after fire management, in both the northern and southern populations (Table 2; Fig 8).

**Fat score:** The early wet season fat score in the northern populations (of all three species) was higher than that of the southern counterpart population before fire management, but similar to that of the southern population after fire management (Table 2; Fig 9).

Model statistics for a) the seasonal change in haematocrit; b) keel score in the wet season; and c) fat score in the early wet season, before and after the imposition of fire management. Each species was modelled separately; the sample size in each analysis is indicated in the relevant column headings. Significant effects are emboldened.

## Discussion

In this study, we found that condition indices among two populations of three seed-eating bird species varied with contrasting fire regimes. We were able to identify the time of year when fire regimes affected condition, and thus shed light on the mechanisms causing decline. Based on this information, we predicted that imposed fire management to reduce fire frequencies, fire size and fire intensity (resulting in an increase in the area of long-unburnt vegetation), in the zone previously exposed to an ‘extreme’ fire regime would cause the condition indices, especially of the most vulnerable species, to ‘normalise’. The results supported the prediction. This study demonstrates how applied management can be used productively to understand ecological relationships in a way that also helps promote population recovery.

### Do extreme fire regimes affect seed-eating birds?

The link between changed fire regimes and population decline in seed-eating savanna birds has previously been based on two indirect lines of evidence. First, distributional changes of seed-eating species has been least pronounced in areas of higher topographical complexity; such areas are probably relatively protected from the impacts of extensive, intense fires and cattle [49] and attributes of the soil surface (i.e. rockier, drier) may slow seed loss due to burial [67]. Second, it has been hypothesised that extensive, intense fires homogenise the spatio-temporal variability in seed production from different grass species, thus limiting foraging options [55, 56].

Our results provide the most direct evidence that grass seed-eating birds are indeed sensitive to the repeated extensive, intense fires that have typified fire regimes across much of northern Australia in recent decades. Condition indices (seasonal haematocrit, keel score and fat score in the wet season) in all three species correlated with fire regime; moreover, when fire in the northern zone of the study area was managed to resemble the more benign regime of the southern zone, the condition indices of all three species converged on the patterns of their southern counterparts. The Gouldian finch, a seed-eating species that has undergone significant contemporary declines in distribution and population size, showed the strongest variation in condition with fire treatment, and the double-barred finch showed the most muted relationship with fire regime.

The syndrome of condition indices associated with a high frequency of extensive, high intensity fires is summarised in Table 3. Extreme fire regimes were associated with high mean haematocrit in the late dry season and high fat scores in the early wet season for all three species; with distinctive seasonal haematocrit profiles and reduced wet season keel scores in long-tailed finches and especially Gouldian finches, and with elevated CORT for Gouldian finches in the wet season. Of the three species, the breeding, feeding and movement patterns of Gouldian and long-tailed finches are most similar (with double-barred finches having a more distinctive ecology [71]), so the relative similarity of condition indices between Gouldian and long-tailed finches (versus double-barred finches) is credible.

**Table 3:** Summary of the condition index patterns of the three finch species.

	Seasonal haematocrit (of north population compared with the south)	Keel—wet season	Fat—early wet season	Residual CORT (Gouldians only)
Gouldian	With extreme fire, mean haematocrit is higher in the late dry season, with a much more marked seasonal decline.	With extreme fire, reduced keel scores in the wet season.	High fat score in the early wet season with extreme fire.	With extreme fire, relative CORT levels increase through seasons (but decreases in the south).
Long-tailed	With extreme fire, mean haematocrit is high with a slight seasonal increase (but it declines seasonally in south).	With extreme fire, reduced keel scores in the wet season.	High fat score in the early wet with extreme fire.	
Double-barred	With extreme fire, mean haematocrit is slightly higher with little seasonal pattern (whereas it declines seasonally in south).	No relationship to fire regime.	High fat score in the early wet with extreme fire.	

The condition syndrome associated with an extreme fire regime is plausibly caused by a reduced food supply throughout the late dry to the end of the wet season. High haematocrit can be caused by high levels of activity [77], which could occur if birds are flying further to find food in the late dry season. High fat scores in the early wet season for northern populations are also consistent with food unpredictability; birds may store more fat when faced with an insecure food supply, despite the potential negative consequences of the extra weight for flight energetics and predator avoidance [78, 79]. However, prolonged food deprivation can lead to loss of muscle mass [80], which could explain the reduced keel scores of northern Gouldian finches and long-tailed finches later in the wet season. Increasing CORT scores in Gouldian finches as the seasons progressed supports the idea that chronic food limitation leads to increased stress responses [81]. One unexplained result is the increase in fat score for southern populations of Gouldian finches and double-barred finches as fire regimes changed; but this is arguably less notable than 1) the consistently different (much higher) fat scores of northern populations of all three species before fire management, and (in contrast) 2) the consistently similar fat scores for northern and southern populations of each species after fire management had begun (regardless of how fat scores in the south had changed).

### Alternative explanations for the condition patterns

This study compared finch populations from just two areas with an initially contrasting fire regime. Thus, treatments were not replicated spatially. However we were able to draw some inference from the north-south population differences among species (declining and non-declining), and inference was further strengthened by examining condition changes over time for each population of each species, as fire regimes changed in the north but not the south. Other than fire regime, we can think of no plausible explanation for the observed difference in finch condition indices between north and south in the earlier years of the study, for the differences in condition indices among species (with Gouldian finches showing the greatest range/sensitivity), and for the convergence of the north-south condition indices in the latter part of the study when fire regimes were harmonised. The only other likely landscape-scale environmental driver is rainfall, but the rainfall patterns in the two Zones were extremely similar. Fires likely affect the finches via seed availability; the alternative possibilities can be discounted: fire could affect finches (in the north, in the earlier years) by reducing the availability of tree hollows, increasing predator density, or by increasing exposure to high temperatures from a reduced canopy. We can exclude changes in the abundance of tree hollows because the population condition response was faster (<5 years) than hollows could re-form. Higher predation risk could cause higher activity levels in the late dry, but the pattern of high fat and then low keel scores in the wet season cannot be explained by predation risk. Increased exposure would likely lead to reduced activity levels and thus haematocrit by finches, opposite to the observed result.

### Seasonal timing of vulnerability for seed-eaters

Most earlier studies have proposed that seed-eaters are most vulnerable to food shortages in the two to four week period of the early wet season, because the onset of rains removes the store of annual seeds that birds use over the dry season before perennials are mature and seeding [49, 54–57, 68] and also because moult (which is being completed then) may confer an extra physiological burden [82]. However, our results suggest that the vulnerability of seed-eaters to food shortages extends across a longer temporal window than just the early wet season, especially for Gouldian finches. The seasonal haematocrit pattern for Gouldian finches exposed to an extreme fire regime differed from the southern population from the late dry to the early wet season (haematocrit higher); this period covers about four months. The seasonal haematocrit pattern of long-tailed finches also changed with fire management, in this case mostly because of a reduction in the early and later wet season haematocrit values; this period covers about two months. The impacts of fire regime on keel scores were most pronounced later in the wet season.

Through the dry season, grass-seed eaters rely on a store of annual grass seed produced at the end of the previous wet season. This store is depleted over each dry season by seed predators, burial, and also by fire [50, 53, 57, 83]. Fires of greater intensity destroy larger proportions of the seed, with intense fires consuming 40% of seed [83] (Legge unpublished data). This reduction in dry season seed stores may cause seed-eating birds to then fly further in search of seed, which would result in higher haematocrits to sustain that elevated activity.

In the wet season, seed-eaters rely on a small number of perennial grass species, consuming the seeds directly off plants as they ripen [54, 55, 68]. Fire affects seed production and survival of grass species in different ways; some species require several fire-free years to reach peak seed production [84]. On the other hand, fire after the first rains can synchronise flowering and increase seed set in one finch food plant, *Alloteropsis semialata* [51] but this fire and rain combination may also create gaps before and after this flush of abundance. At our study site, spinifex *Triodia* spp. was a key resource for Gouldian finches and other seed-eating species in the early wet season. Most of our observations and captures of Gouldian finches during this period were made in patches of *Triodia epactia*, *T*. *bitextura*, *T*. sp. nov. aff. *T*. *schinzii*, all of which require two or more years between fires to produce seed [53, 84, 85]. At the beginning of our study, almost 50% of the northern zone was burnt every year (versus an average of 15% pa in the south), and the extent of long-unburnt vegetation in the north was much less than in the south; with this fire regime, spinifex seed would have been relatively scarce, causing nutritional stress to the birds. As northern fire frequency decreased and the mean age of vegetation increased (Fig 3), spinifex seed availability would have increased. Thus, the condition changes (to haematocrit, keel score, fat score) of Gouldian finches in the wet season could be related to the impacts of reduced fire frequency on the production of spinifex seed.

Gouldian finches would be more strongly affected than long-tailed finches by wet season seed shortages because they are obligate hollow-nesters; by the mid wet season we regularly observed birds defending hollows in preparation for breeding. Favoured hollows are restricted to a very small number of tree species and relatively specific topographical features [61, 70]. Active hollow defence therefore constrains the movements and foraging options of birds to within a certain radius of these hollows. If the hollow is located in an area with limited grass seed production, Gouldian finches can either stay and lose condition, or move and lose their tree hollow. In contrast, other finch species that nest in shrubs and dense grass tussocks as well as hollows, can build their nests nearer favourable foraging areas, and escape the restriction imposed by fixed nest sites. Some individual Gouldian finches captured in the wet season in the north in the early years of the study were emaciated, with very low keel scores and haematocrit values (e.g. >14% below the population mean).

### Using condition as a substitute for population census

We used condition indices rather than population census to track the response of populations to fire regime, mainly because monitoring population persistence or individual survival and breeding in these mobile and relatively short-lived species is impractical [72]. Assessing condition also has the potential advantage of picking up signs of stress before the situation is so extreme that mortality occurs, and remedial management becomes obsolete. Condition measures have been successfully used in many other studies that have assessed habitat quality and the impacts of fragmentation, and have identified stressed populations and the mechanisms of decline (reviewed in [62, 63]). However, although condition can be an important determinant of fitness, condition variation must be interpreted cautiously, because individuals optimise their muscle volume, fat stores, haematocrit and CORT response in relation to many physiological, ecological, social and environmental factors [77, 86–88]. These complexities can make interpreting variation complex and sometimes counter-intuitive. Nevertheless, using a combination of indices that are easily collected in the field (muscle and fat scores) with others that require access to laboratory equipment (haematocrit, CORT concentration), we found variation that sensibly discriminated among species, and among populations exposed to contrasting fire regimes. Assessing a number of separate condition indices improved confidence in interpretation, but seasonal haematocrit variation was the most clearly unique index for distinguishing the threatened Gouldian finch from other finches when exposed to frequent high intensity fires (Figs 5–9).

## Conclusion

This research confirms that the condition of Gouldian finches (and other grass seed-dependent savanna species) can be profoundly affected by fire regime, and that landscape management of fire can moderate that effect. Given the importance of fire in shaping savanna ecology, its presumed role in driving contemporary declines in a range of taxa in the Australian tropical savannas, and a growing body of research on the impacts of fire on biodiversity, this is a rare example of applied research demonstrating positive responses by a population of a threatened species within an adaptive management paradigm [38]. A small number of focal studies on other declining taxa have revealed relationships between species and fire that indicate specific fire management recommendations [54, 89–93]. In addition, a small number of broad fauna monitoring programs have been attached to large-scale fire management [13, 39, 94–96]. However, neither these species-specific studies nor the larger-scale fire manipulations have targeted whether threatened species population recovery arises from the applied management. To some extent, this lacuna exists because of funding and logistic hurdles associated with landscape-scale management experiments. However, coordinated large-scale fire management programs, especially on conservation, pastoral and indigenous tenures, are now developing across northern Australia, sometimes in response to the growing interest in carbon emission abatement. These initiatives provide valuable opportunities not only for carrying out applied research at large scales but also for effecting recovery of species that have otherwise been declining. This study broadly demonstrates the value of the adaptive management model and specifically recommends that a reduction in the frequency, size and intensity of fires in northern Australia will benefit grass seed–eaters, including the threatened Gouldian finch.

## Supporting Information

S1 File Data used for the pre-management condition survey of populations of three finch species experiencing contrasting fire regimes. (XLS)

S2 File Data used for the analysis of condition change in response to fire management. (XLS)
